# Exploring the therapeutic potential of β-nicotinamide mononucleotide (NMN) in regulating canine NT-proBNP levels

**DOI:** 10.5455/javar.2026.m1005

**Published:** 2026-03-05

**Authors:** Zipeng Jiang, Liang Mei, Zhiyi Huang

**Affiliations:** 1 College of Animal Science and Technology & College of Veterinary Medicine, Zhejiang A&F University, Hangzhou 311300, China; 2 South China University of Technology, School of Biology and Biological Engineering, Guangzhou 510641, China; 3 Guangdong VTR Bio-tech Co., Ltd, Zhuhai 519060, China

**Keywords:** Canine heart disease, NT-proBNP, β-Nicotinamide Mononucleotide (NMN), early diagnosis, treatment strategy, NAD^+^

## Abstract

**Objectives:** N-terminal pro–B-type natriuretic peptide (NT-proBNP) is a key biomarker for assessing cardiac function in companion animals and plays a critical role in the diagnosis and management of heart disease. β-Nicotinamide Mononucleotide (NMN), a precursor of NAD^+^, has attracted attention for its potential to enhance mitochondrial function and energy metabolism. This study aimed to evaluate the efficacy of oral NMN supplementation in reducing elevated NT-proBNP levels in dogs and to assess its potential clinical applicability in veterinary practice.

**Materials and Methods:** Dogs with elevated NT-proBNP levels were administered oral NMN at weight-adjusted doses (10–30 mg per dose) twice daily. Treatment continued until NT-proBNP levels returned to the normal reference range. Serum NT-proBNP and symmetric dimethylarginine (SDMA) concentrations were measured, and clinical data were systematically analyzed to evaluate treatment response.

**Results:** NMN supplementation significantly reduced NT-proBNP levels in dogs without evident cardiac structural abnormalities or with mild-to-moderate NT-proBNP elevation. Improvements were particularly notable in cases with preserved cardiac structure and function. However, in dogs with severe heart disease or complex comorbidities, NMN alone demonstrated limited efficacy and required combination with conventional therapeutic interventions to achieve optimal outcomes.

**Conclusions:** Oral NMN administration may serve as a beneficial adjunctive strategy for reducing NT-proBNP levels in dogs without significant structural cardiac defects or advanced functional impairment. Its cardioprotective effects are likely associated with improved myocardial energy metabolism and mitochondrial function. Further controlled clinical studies are warranted to validate its therapeutic role in veterinary cardiology.

## 1. Introduction

Amid a burgeoning emphasis on pet wellness, heart disease has risen to prominence as a critical concern in veterinary medicine, particularly given its prevalence among canine companions. The imperative for early identification and intervention in cases of canine cardiac disorders has catalyzed a surge of research interest and clinical focus. Under physiological equilibrium, cardiomyocytes release pro-B-type natriuretic peptide (proBNP), which subsequently enters the circulatory system. During periods of myocardial strain or ischemia, this precursor is enzymatically split into two peptides: BNP and NT-proBNP [1]. While BNP is rapidly cleared from the bloodstream, NT-proBNP exhibits a significantly longer half-life, accumulating to higher levels within the body [2]. This characteristic renders NT-proBNP a superior biomarker for reflecting cardiac health status. Its elevated blood levels serve as an early sentinel for heart failure and provide a nuanced assessment of cardiac dysfunction, outperforming BNP in diagnostic utility and prognostic value [3].

NT-proBNP, the N-terminal pro-brain natriuretic peptide, is a highly sensitive marker for evaluating cardiac function and is pivotal in the early detection and ongoing management of heart disease in companion animals [4]. Motivated by this significance, our research aims to investigate the potential of β-Nicotinamide Mononucleotide (NMN), a novel oral therapeutic, to modulate elevated NT-proBNP levels in pets.

NMN, as a direct precursor to Nicotinamide Adenine Dinucleotide (NAD^+^), has attracted scientific interest for its ability to enhance mitochondrial function and metabolic efficiency. NMN is fundamental to physiological processes in animal cells, can be synthesized intracellularly, and is obtained from dietary sources [5]. Its biological activities are chiefly mediated through its conversion to NAD^+^, a molecule that is not merely a coenzyme but also a substrate for a multitude of signaling pathways [6]. NAD^+^, also referred to as Coenzyme I or Nicotinamide Adenine Dinucleotide, is involved in a vast array of cellular reactions, supporting cellular vitality and playing a critical role in energy metabolism. A landmark 2016 study by researchers at Washington University School of Medicine illustrated that when mice ingested NMN dissolved in water, the NMN concentration in their bloodstream escalated within 10 min [7]. Within half an hour, NMN had disseminated through the bloodstream to various tissues, where it was swiftly converted into NAD^+^, leading to a significant increase in NAD^+^ levels. This rapid uptake and conversion underscore the potential of NMN as an efficacious means to boost NAD^+^ levels and, by extension, improve mitochondrial health and cellular energy production [6].

Despite demonstrating promise in enhancing cardiac function across human and animal studies, the specific impact of NMN on NT-proBNP levels remains largely unexplored, particularly in veterinary medicine. As a cardinal biomarker of cardiac health, elevated NT-proBNP levels typically signal increased cardiac strain or structural defects [8]. Yet targeted therapies aimed at normalizing these levels are scarce.

This research initiative seeks to fill this therapeutic void by investigating the potential of NMN as a novel treatment strategy for canine heart disease. By focusing on NMN’s ability to regulate NT-proBNP levels, we aim to introduce a convenient, innovative oral therapy. The crux of our innovation hinges on NMN’s dual benefits: Directly enhancing cardiac performance while facilitating early detection and proactive management of cardiac health through NT-proBNP modulation. By administering NMN orally, our study aims to make significant strides in safeguarding cardiac health and controlling NT-proBNP levels. This approach not only advances the field of veterinary cardiology but also paves the way for a safer, more effective treatment regimen that enhances the longevity and quality of life of our beloved pets.

## 2. Materials and Methods

### 2.1. Ethical approval

The experimental procedures were conducted strictly in accordance with guidelines provided by the Animal Welfare Committee, and the protocol was approved by the Animal Care and Use Committee of Guangdong VTR Biotech (approval number 2021307), Zhuhai, China.

### 2.2. Experimental material

The dietary regimen was formulated to meet the nutritional requirements outlined by the Association of American Feed Control Officials. Prior to the trial, all necessary vaccinations and deworming procedures were carried out, and none of the animals received any medications known to alter gut microbiota, such as antibiotics, within a month preceding the study. Daily cleaning and disinfection routines were implemented to uphold a clean environment within the animal enclosures. Each animal receives a daily ration of food, and those with pre-existing conditions are managed and exercised according to veterinarians’ instructions. Throughout the experimental period, all animals had free access to clean drinking water. Nicotinamide mononucleotide (NMN, CAS: 1094-61-7, purity > 99.5%) was supported by Guangdong VTR Bio-tech Co. Ltd.

### 2.3. Animals and experimental design

The study encompassed eight canine subjects exhibiting abnormal NT-proBNP levels, representing a spectrum of ages, genders, weights, and clinical manifestations. This diverse cohort comprised asymptomatic dogs with NT-proBNP elevations as well as those diagnosed with severe cardiac disease or chronic kidney disease. All animals were orally administered a NMN-based formulation, with individualized doses set within a range of 10–30 mg per dose, given twice daily, adjusted according to their body weight and the NT-proBNP levels (The application range for small dogs is 10–20 mg per dose, and the application range for large dogs is 20–30 mg per dose). Throughout the 3-month intervention period, the medication was discontinued once NT-proBNP levels normalized, and NT-proBNP levels were reevaluated every 2–3 weeks to monitor treatment response. Document the changes in NT-proBNP levels before and after the treatment. NT-proBNP levels were found to be abnormally elevated during routine health checks or clinical examinations of eight dogs. The group comprised individuals both without noticeable clinical signs and those exhibiting overt heart disease alongside other underlying health conditions. The information about the dogs involved in the trial is shown in Table 1.

**Table 1.** Basic situation of experimental dogs.

Dog No.	Breed	Age (years)	Gender	Weight (kg)	Clinical Symptoms
1	Poodle	9	Female	2.8	Occasional diarrhea, dental calculus, elevated NT-proBNP pre-surgery
2	Golden Retriever	6	Female	28	Elevated NT-proBNP on routine check, otherwise asymptomatic
3	Border Collie	12	Female	14.7	Abnormal NT-proBNP detected during health check
4	Poodle	12	Female	6.3	Polycythemia, allergic dermatitis
5	Mixed breed	13	Female	4.4	Coughing, fainting spells
6	Cavalier King Charles Spaniel	15	Male	9.1	Degenerative heart disease, pulmonary edema, enlarged left atrium
7	Shih Tzu	18	Male	4.8	Advanced heart disease, showing signs of heart failure, abnormal NT-proBNP
8	Samoyed	15	Male	14.5	Chronic kidney disease

### 2.4. Sample collection and analysis

The sample collection was conducted as in the previous study [9]. Briefly, after one week of NMN treatment and an overnight fast, blood samples were collected from all dogs via the forelimb vein. The samples were allowed to rest for 30 min before being centrifuged at 3500×*g* at room temperature (20–25°C) for 15 min. The supernatants obtained after centrifugation were aliquoted into microcentrifuge tubes and stored at –80°C for future analysis. Serum levels of NT-proBNP and SDMA were determined using a commercial ELISA kit from ELK Biotechnology Co., Ltd., Wuhan, Hubei, China. All experimental procedures were conducted strictly according to the instructions provided.

### 2.5. Statistical analysis

All data were recorded with Excel software (Microsoft Inc, Washington DC, USA). Data was analyzed by SPSS 22.0 software (IBM company, New York, USA). To compare NT-proBNP levels before and after NMN treatment within the same group, a paired t-test was used. *p*-value < 0.05 was considered statistically significant. Data are expressed as the mean. GraphPad Prism version 9.0 (San Diego, CA, USA) was used to visualize the data.

## 3. Results

### 3.1. Animals’ status

As detailed in Table 1, eight canine patients were enrolled to assess their NT-proBNP levels following standard physical evaluations. Here are brief summaries of each case:

Dog 1: A 9-year-old, 2.8 kg female Poodle presented with intermittent episodes of diarrhea and dental tartar during her physical exam. An elevated NT-proBNP level was noted during her preoperative evaluation.

Dog 2: A 6-year-old, 28.5 kg female Golden Retriever showed an increased NT-proBNP level during her routine health screening, with no other notable abnormalities identified in ancillary tests.

Dog 3: A 12-year-old, 14.7 kg female Border Collie with a longstanding history of chronic hip arthritis was found to have elevated NT-proBNP and SDMA levels during her medical assessment.

Dog 4: A 12-year-old, 6.3 kg female Poodle diagnosed with polycythemia and allergic dermatitis revealed abnormal NT-proBNP levels during her health check-up.

Dog 5: A 13-year-old, 4.4 kg female mixed-breed dog displayed heart-related symptoms such as coughing, dyspnea, and syncope, coinciding with an abnormal NT-proBNP reading.

Dog 6: A 15-year-old, 9.1 kg female Cavalier King Charles Spaniel, suffering from advanced chronic degenerative heart disease, manifested symptoms of heart failure including syncope and seizures, alongside elevated NT-proBNP levels.

Dog 7: An 18-year-old, 4.8 kg male Shih Tzu, grappling with severe degenerative heart disease, showed symptoms such as coughing, renal failure, and signs of congestive heart failure.

Dog 8: A 15-year-old, 28.5 kg female Samoyed with a documented history of chronic kidney disease had her routine health examination reveal elevated NT-proBNP and SDMA levels.

These summaries highlight the varied clinical presentations and comorbidities in dogs with elevated NT-proBNP levels, underscoring the importance of comprehensive veterinary assessments.

### 3.2. NMN alleviates abnormal NT-proBNP level

**Figure 1.**
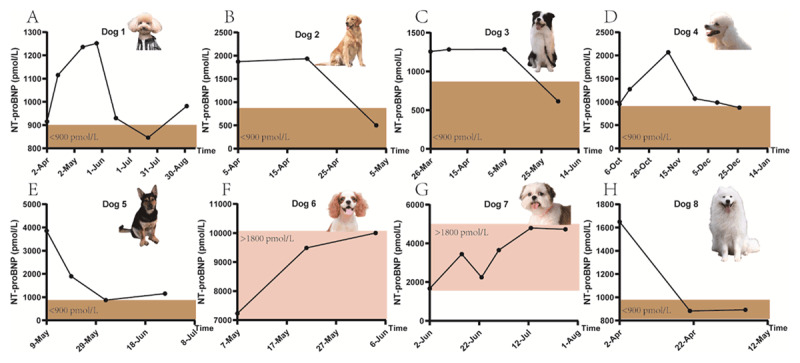
Eight dogs and the changes of NT-proBNP levels during the experimental period.

Within the scope of this investigation, eight dogs with elevated NT-proBNP levels were administered oral NMN, yielding a spectrum of therapeutic outcomes. A graphic representation of the comprehensive health status of all canine participants is depicted in Figure 1.

For Dog 1 and Dog 2, who presented with no overt comorbidities and exhibited mild-to-moderate NT-proBNP elevations, consistent NMN supplementation resulted in a decline in NT-proBNP concentrations, achieving levels below the normal range. Initially, Dog 1 was prescribed NMN at a dose of 10 mg per dose, administered twice daily, for a duration of one month.

Subsequent monitoring revealed a marginal increase in NT-proBNP levels relative to baseline. In response, the NMN dosage was doubled, and treatment was extended for an additional 1.5 months. This adjustment led to NT-proBNP levels returning to within the normal range. However, discontinuing the medication for about ten days precipitated a recurrence of elevated NT-proBNP levels, as recorded in Table 2. Dog 2 responded favorably to NMN therapy, with NT-proBNP levels normalizing after just two weeks of oral administration, as documented in Table 3.

**Table 2.** NMN Administration and NT-proBNP Changes in Dog 1.

Dog 1	A 9-year-old, 2.8 kg female Poodle was identified with abnormal NT-proBNP levels during a routine health examination and subsequently administered NMN orally as the sole intervention.						
Time of taking NMN	4.2	4.14	5.11	5.26	6.16	7.21	9.1
NT-proBNP (pmol/l)	915	1115	1236	1251.99	929.6	845.8	981.6
Medication dose	–	10 mg bid	20 mg bid			–	–

Dog 1, a 9-year-old, 2.8 kg female Poodle, was administered NMN at 10 mg bid, later increased to 20 mg bid due to rising NT-proBNP levels. NT-proBNP levels fluctuated initially but returned to normal after dosage adjustment.

**Table 3.** NMN Administration and NT-proBNP Changes in Dog 2.

Dog 2	A 6-year-old, 28.5 kg female Golden Retriever experienced elevated NT-proBNP levels upon examination and was exclusively treated with oral NMN.		
Time of taking NMN	4.5	4.19	5.3
NT-proBNP (pmol/l)	1871.6	1934	< 500
Medication dose	–	30 mg bid	-

Dog 2, a 6-year-old, 28.5 kg female Golden Retriever, received NMN at a dose of 30 mg bid. NT-proBNP levels normalized within two weeks of treatment.

Dogs 3 and 4, despite their chronic conditions, showed no physical anomalies during examination, nor were any organic heart alterations observed. Both dogs were treated with NMN at a dose of 20 mg bid for approximately 2.5 months. Reassessments revealed an initial spike in NT-proBNP levels, which subsequently normalized, as outlined in Tables 4 and 5.

**Table 4.** NMN Administration and NT-proBNP Changes in Dog 3.

Dog 3	A 12-year-old, 14.7 kg female Border Collie with a history of chronic hip arthritis had abnormal NT-proBNP and SDMA levels detected during a medical checkup and was managed solely through oral NMN administration.			
Time of taking NMN	3.26	4.5	5.5	6.3
NT-proBNP (pmol/l)	1257	1284	1284.9	614.3
Medication dose	20 mg bid	20 mg bid	20 mg bid	–
SDMA (ng/ml)	18	18	17	14

Dog 3, a 12-year-old, 14.7 kg female Border Collie with chronic hip arthritis, was given NMN at 20 mg bid. Despite initial increases, NT-proBNP levels eventually normalized.

**Table 5.** NMN Administration and NT-proBNP Changes in Dog 4.

Dog 4	A 12-year-old, 6.3 kg female Poodle with polycythemia and allergic dermatitis was found to have abnormal NT-proBNP levels during a health check. After observing for one week with no improvement, NT-proBNP levels continued to rise, the decision was made to initiate oral NMN therapy without additional medications.					
Time of taking NMN	10.6	10.13	11.08	11.26	12.11	12.26
NT-proBNP (pmol/l)	953.2	1273.7	2068.1	1071	987	876
Medication dose	20 mg bid					

Dog 4, a 12-year-old, 6.3 kg female Poodle with polycythemia and allergic dermatitis, was administered NMN at a dose of 20 mg bid. NT-proBNP levels showed an initial rise followed by a decline.

Dogs 5, 6, and 7, afflicted with severe cardiac disorders, manifested markedly high NT-proBNP levels alongside symptoms indicative of heart failure, such as fainting and coughing. Alongside conventional heart disease medications, oral NMN supplementation was initiated. Dog 5 responded positively to NMN, exhibiting a notable reduction in NT-proBNP levels throughout the treatment phase, as summarized in Table 6. Conversely, Dogs 6 and 7 experienced a persistent escalation in NT-proBNP levels that persisted until their demise, as recorded in Table 7 and Table 8, respectively.

**Table 6.** NMN Administration and NT-proBNP Changes in Dog 5.

Dog 5	A 13-year-old, 4.4 kg female mixed-breed dog suffering from heart-related coughing, breathlessness, and fainting spells, alongside abnormal NT-proBNP levels, was prescribed a regimen that included oral NMN along with complementary cardiovascular medications.			
Time of taking NMN	5.9	5.19	6.2	6.26
NT-proBNP (pmol/l)	3856	1897.3	871	1149.7
Medication dose	20 mg bid			

Dog 5, a 13-year-old, 4.4 kg female mixed-breed dog with heart-related symptoms, was treated with NMN at a dose of 20 mg bid alongside other cardiovascular medications. NT-proBNP levels decreased noticeably during treatment.

**Table 7.** NMN Administration and NT-proBNP Changes in Dog 6.

Dog 6	A 15-year-old, 9.1 kg female Cavalier King Charles Spaniel, afflicted with advanced chronic degenerative heart disease, experienced symptoms such as fainting and seizures indicative of heart failure, coupled with abnormal NT-proBNP levels. The management plan comprised oral NMN in addition to a tailored regimen of other cardiac therapies.			
Time of taking NMN	5.7	5.21	6.4	7.5
NT-proBNP (pmol/l)	7231.5	9486.4	10000	–
Medication dose	20 mg bid			Dead

Dog 6, a 15-year-old, 9.1 kg female Cavalier King Charles Spaniel with advanced chronic degenerative heart disease, was given NMN at a dose of 20 mg bid in addition to other cardiac therapies. NT-proBNP levels continued to rise until the dog’s death.

**Table 8.** NMN Administration and NT-proBNP Changes in Dog 7.

Dog 7	An 18-year-old, 4.8 kg male Shih Tzu with severe degenerative heart disease, exhibiting symptoms including coughing, renal failure, and congestive heart failure, was treated with oral NMN alongside other cardiac medications. Unfortunately, after a period of one and a half months, the dog succumbed to heart failure.					
Time of taking NMN	6.2	6.15	6.23	6.30	7.13	7.27
NT-proBNP (pmol/l)	1669.5	3444.9	2246	3648.6	4790	4724
Medication dose	20 mg bid					Dead

Dog 7, an 18-year-old, 4.8 kg male Shih Tzu with severe degenerative heart disease, received NMN at a dose of 20 mg bid along with other cardiac medications. NT-proBNP levels fluctuated, and the dog eventually succumbed to heart failure.

Dog 8, diagnosed with chronic kidney disease and undergoing long-term subcutaneous fluid therapy and oral medication for renal management, presented with an unexpected surge in NT-proBNP levels during routine monitoring, with no apparent organic heart alterations. Upon initiating NMN therapy at a dose of 30 mg bid, sustained over a month, a reevaluation 20 days post-initiation revealed normalized NT-proBNP levels. These levels remained stable within the normal range until the dog’s untimely passing due to acute gastric dilation, as documented in Table 9.

**Table 9.** NMN Administration and NT-proBNP Changes in Dog 8.

Dog 8	A 15-year-old, 28.5 kg female Samoyed with a history of chronic kidney disease underwent a routine health check where elevated NT-proBNP and symmetric dimethylarginine (SDMA) levels were detected. The treatment plan consisted of regular fluid therapy and oral NMN administration. Sadly, on May 7th, the dog passed away unexpectedly due to a gastric dilatation event.			
Time of taking NMN	4.2	4.21	5.6	5.7
NT-proBNP (pmol/l)	1649.6	882.6	892	–
Medication dose	30 mg bid			Dead
SDMA (ng/ml)	24	18.2	17.9	–

Dog 8, a 15-year-old, 28.5 kg female Samoyed with chronic kidney disease, was administered NMN at 30 mg bid. NT-proBNP levels normalized but the dog passed away due to acute gastric dilation.

A holistic review of the data suggests that NMN therapy is efficacious in dogs without substantial cardiac structural defects or those displaying mild-to-moderate elevations in NT-proBNP. However, in cases of severe heart disease or when complicated by additional health issues, the benefits of NMN may necessitate augmentation with conventional treatment strategies.

## 4. Discussion

The measurement of NT-proBNP levels stands as a pivotal diagnostic tool in identifying heart disease in dogs. It excels in differentiating between cardiac and non-cardiac etiologies for symptoms such as respiratory difficulty, reduced exercise tolerance, or persistent coughing. Coupled with echocardiographic findings, NT-proBNP serves as a crucial biomarker for monitoring disease progression and early detection of cardiac anomalies [8].

A plasma NT-proBNP concentration below 900 pmol/l in dogs signals a diminished probability that the current manifestation of respiratory distress, exercise intolerance, or cough is rooted in cardiac pathology. Levels ranging from 900 to 1800 pmol/l hint at a possible cardiac origin for these symptoms but also leave room for alternative diagnoses, thus requiring further investigative steps to ascertain the exact cause. Conversely, a concentration exceeding 1800 pmol/l strongly suggests cardiac involvement, warranting a more comprehensive cardiac workup to confirm the diagnosis and guide appropriate therapeutic interventions [10].

During our study, we monitored and treated eight dogs with elevated NT-proBNP levels. Among them, Dogs 1 and 2 initially displayed mild-to-moderate increases in NT-proBNP concentrations, which further escalated upon reassessment two weeks later. Following sustained oral NMN supplementation, both dogs showed a decline in NT-proBNP levels, achieving values below the 900 pmol/l threshold. Notably, neither dog exhibited any overt clinical symptoms; the elevated NT-proBNP levels were incidentally discovered during routine health screenings, with plasma levels exceeding 900 pmol/l. Comprehensive evaluations by echocardiography and electrocardiogram (ECG) did not reveal any significant abnormalities in cardiac function or structure, suggesting that the elevated NT-proBNP might have been an early indicator of subclinical cardiac stress. One study verified that the NAD^+^ content could reduce the aspects of striated muscle disease in a dog [11]. Another research detected the positive effects of NMN in prevent obesity in female mice [12]. All this evidence demonstrated that NMN is useful for animal health, and the present study showed clear positive effects on NT-proBNP levels in dogs.

The results are like the previous studies which identified that the NAD^+^ precursor NMN had the potential against cardiac aging and anti-aging function [13]. Similarly, dogs 3 and 4 exemplify the potential of oral NMN supplementation to address elevated NT-proBNP levels, particularly in canines with chronic inflammation without manifest cardiac structural impairment. Continuous NMN administration effectively lowered plasma NT-proBNP concentrations below the 900 pmol/l threshold, underscoring the therapeutic utility of NMN in this context.

Dog 8’s case notably expands the scope of NMN’s application beyond cardiac conditions. This dog, suffering from chronic kidney disease, exhibited elevated NT-proBNP levels, a phenomenon not directly attributable to renal pathology but potentially influenced by impaired kidney function affecting NT-proBNP clearance [14]. The successful reduction of NT-proBNP levels through NMN administration in Dog 8 highlights the broader potential of NMN in maintaining multi-system health, including its beneficial effects in non-cardiac conditions.

Our findings corroborate recent research, which posits that NMN enhances energy generation in cardiac muscle cells, eases cardiac workload [15]. NMN exerts cardioprotective effects through three interconnected NAD^+^-dependent pathways: By elevating intracellular NAD^+^ levels, NMN activates SIRT1 deacetylase which- (i) Inhibits FOXO3a-mediated transcription of pro-apoptotic Bim, attenuating cardiomyocyte apoptosis, while concurrently modulating FOXO1-dependent oxidative stress responses to reduce hypoxia-induced inflammation; (ii) Sustains genomic stability during ischemic insults through PARP-1-dependent DNA repair mechanisms facilitated by the PARP-NAD^+^-SIRT axis, preventing cumulative DNA damage in cardiomyocytes; and (iii) Compensates for pressure overload-induced cardiac stress via monocyte-derived eNampt upregulation – this systemic NMN surge preserves myocardial NAD^+^ pools despite local iNampt downregulation, thereby maintaining cardiac contractility and preventing decompensation.

Collectively, these mechanisms enhance cellular viability by simultaneously mitigating oxidative stress, repairing subcellular damage, and optimizing energy homeostasis under hemodynamic stress. NMN also protects against high-fat diet-induced atherosclerosis in mice and reduces aortic inflammation and oxidative stress [16]. Additionally, other research found that NMN is a potentially effective natural compound for treating insulin resistance [17]. In the relevant research on human or animal use for cardiac aging, Wei et al. [13] describe that, as a nutraceutical, it has anti-aging and cardioprotective potential in animal studies. We observed that NMN has the potential to lower NT-proBNP levels in dogs without detectable cardiac dysfunction or structural anomalies, as well as in those with systemic diseases. Abdellatif et.al discussed that the NMN, NAD^+^ metabolism in cardiac health aging and disease, they demonstrated that the patients with hitherto intractable cardiac diseases, such as heart failure with preserved ejection fraction, may profit from the administration of NAD^+^ precursors [18]. Dog 5’s case highlights that in dogs with cardiac problems yet without pronounced heart failure, NMN’s oral administration in tandem with conventional cardiovascular medications can effectively curtail NT-proBNP levels. This synergy suggests that NMN may enhance the efficacy of standard medications in mitigating heart-related conditions.

However, Dogs 6 and 7’s outcomes suggest that in cases of severe heart disease, NMN’s solitary use, even when complemented by typical heart disease therapy, might not be adequate to fully arrest disease progression. One study presented that NT-proBNP can distinguish congestive heart failure (CHF) from primary respiratory disease in cats with respiratory signs with approximately 90% diagnostic accuracy [19]. In instances of heart failure marked by conspicuous cardiac structural distortions and diminished heart function, NT-proBNP levels are usually substantially elevated. Oral NMN administration might not yield a marked effect on these levels. Additional clinical evidence is needed to validate these observations and explore NMN’s potential role in severe heart disease. This could be attributed to the notion that, in cases of severe cardiac dysfunction, improving energy metabolism alone may not be sufficient to fully restore heart function. Hence, in such severe cases, NMN may require integration with other therapeutic strategies to achieve optimal treatment outcomes.

Moreover, this study underscores the importance of customized treatment protocols, which necessitate adjusting NMN dosages and treatment durations to the unique circumstances of individual pets. Other research used personalized NMN supplementation to monitor NAD levels, tailoring dosage regimens, and optimizing NMN utilization [20]. This approach resonates with the personalized medical strategies endorsed by Zhang et al. [21], which advocate for treatment plans tailored to individual variations to maximize therapeutic benefits while minimizing adverse effects.

## 5. Conclusions

The outcomes of our experimental study indicate that NMN, administered at a dose of 20–30 mg twice daily, holds promise for mitigating plasma NT-proBNP levels in dogs without pronounced cardiac structural anomalies or significant deterioration in heart function. However, NMN seems less effective in markedly lowering plasma NT-proBNP levels in dogs presenting with overt manifestations of heart failure. To summarize, our research underscores the potential of NMN as a therapeutic agent for managing abnormal NT-proBNP levels in dogs. Beyond validating its application, our findings open up new avenues for future investigations. These include a deeper exploration of the molecular pathways through which NMN exerts its effects under diverse cardiac pathophysiological conditions. Moreover, understanding how NMN can complement existing therapeutic interventions will be crucial in formulating more refined and effective treatment protocols.

## Data Availability

The data presented in this study are available from the corresponding author upon reasonable request.

## List of abbreviations

bid, twice daily; BNP, B-type natriuretic peptide; ECG, Electrocardiogram; ELISA, Enzyme-linked immunosorbent assay; eNampt, Extracellular nicotinamide phosphoribosyltransferase; FOXO1, Forkhead box protein o1; FOXO3a, Forkhead box protein o3a; *g*, Relative centrifugal force; iNampt, Intracellular nicotinamide phosphoribosyltransferase; kg, kilogram; mg, milligram; min, minute; NAD^+^, Nicotinamide adenine dinucleotide; ng/ml, nanograms per milliliter; NMN, β-nicotinamide mononucleotide; NT-proBNP, N-terminal pro-brain natriuretic peptide; PARP-1, Poly (ADP-ribose) polymerase 1; pmol/l, picomoles per liter; proBNP, Pro-B-type natriuretic peptide; SDMA, Symmetric dimethylarginine; SIRT1, Sirtuin 1; °C, degrees Celsius.
